# Broad spectrum micronutrients: a potential key player to address emotional dysregulation

**DOI:** 10.3389/frcha.2023.1295635

**Published:** 2023-12-15

**Authors:** Amelia Villagomez, Michelle Cross, Noshene Ranjbar

**Affiliations:** ^1^ Department of Psychiatry, University of Arizona, Tucson, AZ, United States; ^2^ Department of Psychiatry, University of Vermont, Burlington, VT, United States

**Keywords:** nutritional psychiatry, nutrition, integrative medicine (IM), integrative psychiatry, broad spectrum micronutrients, supplements, ADHD treatment, nutraceuticals

## Abstract

Psychiatric conditions are inherently multifactorial and must be understood and addressed within a multidimensional framework. Adequate nutritional intake is necessary for optimal mental health and is thus an essential component of any psychiatric treatment plan; this is especially true as many patients have a diet high in ultra-processed foods. However, due to a variety of factors such as individual biological and behavioral contributors, modern farming practices, and climate change, implementing a healthy diet alone may not be sufficient to satisfy nutritional requirements. Research studies on three formulations of broad-spectrum micronutrients (BSMs) have demonstrated significant efficacy in treating a range of mental health disorders. In particular, outcomes associated with emotional regulation via BSMs across a variety of psychiatric illnesses (ADHD, autism, trauma, mood disorders, nicotine dependence, and psychosis) to date have been positive.

## Introduction

Children and adolescents in the United States are experiencing an unprecedented mental health crisis, which was accelerated by, but cannot be wholly attributed to, the COVID19 Pandemic. The CDC's Youth Risk Behavior Survey Data Summary & Trends Report: 2011- 2021 found that mental health among students has been steadily declining over the past decade (1). A reported 57% of female students and 29% of male students in 2021 expressed persistent feelings of sadness or hopelessness in 2021, up from 36% and 21% the decade prior. Additionally, 30% of female students and 14% of male students reported having seriously considered attempting suicide in 2021, up from 19% and 13% respectively the decade prior (2). Since our current system of care is, by the numbers, not sufficient, we must continue to consider and evaluate all potentially positive contributors to health and wellbeing.

A decade ago, a national survey found that approximately 12% of all children and adolescents and 50% of children and adolescents with chronic illness were reported to be using complementary and alternative therapies. Among these individuals, complementary approaches were most commonly used for colds, musculoskeletal pain, ADHD, and anxiety/stress (3). One may postulate that this points to a recognition in families of the need for more holistic and comprehensive interventions. It is thus critical that clinicians become familiar with complementary and alternative approaches so they may appropriately counsel patients on their safety and efficacy. With the rising popularity of complementary and alternative treatments by patients, physicians are increasingly seeking out education on how to provide appropriate guidance (4).

Proper nutrition is often intuitively accepted by clinicians and patients alike as essential to a healthy life. Research has indeed shown an association between a nutrient-dense diet and better mental health (5–8). Additionally, some studies have shown a correlation between a diet of nutrient-poor ultra-processed foods and the later onset of poor mental health, specifically, depression and anxiety (9–11). Minerals and vitamins are fundamental to maintaining a healthy brain as they support the brain's metabolic functioning, can act as anti-inflammatory agents, are involved in neurodevelopment, modulate genetic expression and cell signaling, and are involved in the production of neurotransmitters and in maintaining biochemical normalcy (12).

Can we obtain the necessary vitamins and minerals from our diet alone? It has been postulated that for some subset of individuals the answer is in fact no. The explanation is tied to both environmental and biological processes. Research across the globe indicates that there have been decreases in the concentrations of certain micro and macronutrients in crops over the last 70–150 years (13–15). Contributing factors include certain farming practices such as prioritizing yield over nutrient density or flavor (13), over tilling of the soil while fertilizing it with only limited essential nutrients (16), and the use of the herbicide glyphosate which may impair absorption of certain minerals such as iron, manganese and nickel (17) while further decreasing soil health (18). There is also concern that the ever-increasing concentration of carbon dioxide in the environment is leading to decreased nutrient density in crops; higher levels of CO2 appear to promote higher concentrations of carbohydrates with lower proportions of protein and micronutrients (15, 19, 20). Furthermore, some people have a congenital need for more than the typical amount of cofactors for optimal enzymatic activity. This may be explained, at least in part, by variation in genetic polymorphisms (21, 22).

### What is a broad-spectrum micronutrient formulation and its potential clinical use based on current research?

The physiologic processes that underlie our mental health and wellbeing require a broad range of micronutrients working in concert. Earlier nutritional research efforts focused primarily on the impact and treatment of single micronutrient deficiencies (e.g., pellagra, scurvy, or Wernicke–Korsakoff Syndrome). Although deficiencies in some micronutrients have been reliably correlated with poorer mental health outcomes, in many cases when single deficiencies are corrected with mono-supplementation there are small to no effects shown in the literature on mental health and wellbeing (22). It can thus be postulated that the underlying issue is not dependent on a single nutrient deficiency, but rather that the entire system needs to be supplemented with a wide range of micronutrients. Popper (22) defines a broad-spectrum micronutrient (BSM) as a formulation or intervention that includes at least 10 different vitamins or minerals.

To date, independent scientists have studied the effectiveness of three commercially available BSM formulas: EMPowerPlus and its variations (https://www.truehope.com/), Daily Essential Nutrients (DEN) and its variations (https://www.hardynutritionals.com/), and the Autism Nutrition Research Center (ANRC) Essentials developed by Arizona State University (https://www.autismnrc.org/). We use the term “variations” to refer to the fact that over time the manufacturer has made slight modifications to their formula. Each formula has approximately 30 minerals and vitamins, along with selected amino acids and antioxidants. Each formula has also been shown in several studies, including RCTs, to affect mental health outcomes (23), which we will summarize here.

### ADHD

Although the evidence supporting the efficacy of the current ADHD medications for core symptoms of ADHD is strong (24), addressing the often-comorbid emotional dysregulation of ADHD continues to be a challenge. Additionally, stimulant treatments may cause undesired side effects, such as worsening of emotional lability, anxiety (25), and appetite suppression/poor nutritional intake. Further complications include the existence of a notable subset of children with a variable and incomplete response to our current standards of treatment for ADHD, as well as the ongoing issues regarding the potential for misuse and/or diversion of stimulant medications. For example, in the Multimodal Treatment of Attention Deficit Hyperactivity Disorder Study (MTA) study, only 68% of participants who received both stimulant medication and behavioral therapy were considered successfully treated (26).

Early, open label studies were the first to demonstrate the potential use of BSM in ADHD showing improvements in core ADHD symptoms and/or associated behavioral dysregulation (27–29). More recently, given the very promising data from open label studies, high quality, randomized placebo-controlled trials of BSM have been conducted for ADHD. The first RCT in children included 93 medication-free 7–12-year-olds who were randomly assigned to either a group that received BSM or placebo capsules for a period of 10 weeks. Core ADHD symptom improvement was modest or low/not significant from placebo; however, BSMs had significant effects on measures of global functioning (CGI-I ES = 0.46; C-GAS ES = 0.48), inattention (CGI-I ADHD ES = 0.53), emotion regulation and aggression (CGI-I Mood ES = 0.51; Parent SDQ ES = 0.52; Teacher BRIEF—Emotional Control Subscale ES = 0.66) (30).

An additional analysis was conducted in this RCT to investigate possible predictors (e.g., serum nutrient status, sex, IQ, MTHFR genetic status) of treatment response across outcomes. There were no consistent predictors of positive response (31). A subsequent open label extension phase followed this group for 1 year. About 80% of those who continued BSMs were labeled as being in remission from their ADHD symptoms vs. 40% of those on medications and 20% of those who stopped all treatment. There were continued symptom improvements over time (32). The improvements observed in children who received BSMs in the short term were sustained during the 1-year follow-up period, and no adverse effects were noted. Similar results were also seen in a naturalistic follow-up study of adults with ADHD (33).

In a multicenter RCT involving 126 children aged 6–12 diagnosed with ADHD, significant improvements were exhibited by those who received the BSM (34). Change was assessed using clinician-rated Clinical Global Impression-Improvement (CGI-I) scales, revealing a significant outcome (*p* < .001, RR = 2.97). Treatment responders, defined as those showing “much” or “very much improved” status according to the CGI-I scale, constituted 54% of the micronutrient-treated group, whereas only 18% of the placebo group were responders. Additionally, those in the micronutrient group grew an average of 6 mm more than those in the placebo group (*p* = .002, *d* = 1.15), not attributable to rebound from previous stimulant use. No serious adverse events or changes in blood/urine tests were found. This trial replicated safety and efficacy data of 2 other RCTs using a similar formula for patients with ADHD (30, 35).

These studies suggest that BSMs are very promising for the treatment of ADHD, particularly in those with emotional dysregulation.

### Aggression

Aggression and rule breaking behavior, which often has an inherently complicated etiology for any one child, is a common presenting concern to child psychiatry clinics and ERs. There have been some investigations into the utility of using vitamin/mineral supplements to assist in treating these behaviors. The first RCT to consider this question involved 62 incarcerated youths ages 13–17. All were encouraged to improve their diet, but a subset was randomized to receive an additional broad spectrum supplement (11 minerals, 12 vitamins). Violence, non-violent rule violations, and total rule violations fell to a significant degree in the BSM group; the effect was even greater when compared to those in the placebo group who did not improve their diet (36).

Eighty schoolchildren aged 6–12 were studied in a RCT for 1 year to evaluate the effects of BSM on rule-breaking behavior (threats, fighting, vandalism, defiance, endangering others, disorderly conduct). It was found that those randomized to the BSM group displayed 47% fewer of these behaviors requiring discipline than those who received placebo (37).

Overall, there appears to be a clear relationship between vitamin/mineral intake and aggression (38). This could have significant ramifications for individuals and our communities; particularly in communities that do not have access to high quality, nutritious food.

### Anxiety and stress

It has been postulated that stress and anxiety, in the activation of the fight or flight response, may increase micronutrient depletion and thus prolong the stress response. Initial outcomes regarding micronutrient supplementation in the reduction of stress and anxiety appear promising. In the open label extended phase of the ADHD study (29), parents reported decreased levels of anxiety in the children who continued BSMs. An open label trial of 17 children ages 8–11 investigating the effect of BSM on stress/anxiety following an earthquake reduced children's post-disaster anxiety by a clinically significant degree (39). Given the urgent need for better pediatric treatment of trauma and promising data in adults, further studies are needed in children to evaluate the effect of BSMs on stress, anxiety and PTSD. In adults, two unblinded RCTs investigated the effects of BSM supplementation on acute post-natural disaster stressors (flood, earthquake) compared to an active comparison (40, 41). The treatment duration ranged from 4 to 6 weeks. Rucklidge et al. (41) studied 91 adults with elevated depression, anxiety, or stress symptoms after the 2011 Christchurch earthquake; participants received either 4 or 8 capsules daily of EMP+ or a B complex as the active comparison. Kaplan et al. (40) studied 56 adults with similar symptoms after the 2013 floods in Alberta, Canada, comparing EMP+ with a B complex and vitamin D as active comparisons. Both studies used the Depression Anxiety and Stress Scale (DASS) as the primary outcome measure. Both studies reported significant reductions in symptoms within all treatment groups. In the Rucklidge study, high-dose EMP+ showed greater clinical improvement in mood, energy and anxiety compared to the B complex control. Kaplan's study found that both EMP+ and B complex were more effective than vitamin D in reducing anxiety and stress symptoms.

### Autism spectrum disorders

Studies in those with autism show promising results for potential improvements in cognitive, emotional, and behavioral domains. A case control study of 44 people with autism ages 2–28 who were given BSM or an equivalent were compared to a matched group of 44 similar individuals who sought treatment as usual. Both groups showed improvement; however, the BSM group had a significantly larger decrease in scores on the Total Aberrant Behavior Checklist, self-injurious behavior intensity, and in improvements on the Clinical Global Impressions scale. The BSM group also reported fewer adverse events as compared to the medication as usual group (42).

Researchers at Arizona State University have been developing a BSM specifically for autism. A randomized placebo controlled 3-month study in children and adults with autism on an earlier version of the supplement showed improvements over placebo in the areas of hyperactivity (*p* = 0.003) and tantruming (*p* = 0.009). The data suggested a possible improvement overall (*p* = 0.02) as well as in receptive language (*p* = 0.03). Nonsignificant correlations were found in expressive language, play, cognition, GI, sleep, sociability (43). An open label research survey was conducted by the same researchers on their current formulation of the BSM. They found improvements over placebo (comparing survey participants to the placebo group of the previously mentioned RCT) by Parent Global Impressions of Autism with an estimated effect size of 0.66. When compared to data collected from the National Survey on Treatment Effectiveness for Autism, the overall benefit scores of this study's respondents were higher than the average score of 28 psychiatric and seizure medications as well as the average score of 58 nutraceuticals. They also found that the BSM respondents had an overall low adverse effect score, which was slightly higher or comparable to the national data on nutraceuticals and much lower than that of the psychiatric/seizure medication group (44).

### Mood disorders

To date, there is limited high quality research on the effect of BSM on mood disorders in children. A database analysis of parent reports of 120 children ages 7–18 showed 46% of the sample improved by at least 50% (*p* < .001, ES = 0.78), 35% of the sample improved by less than 50% and 19% of the sample had symptom worsening. For those who were treatment responders, the number of psychiatric medications used decreased by 74%. These data were collected by the manufacturer but independently analyzed by researchers (45). A case series of 3 children noted improvements in mood after 12 weeks of treatment with BSM by parent and child reports (46).

An open label case series of adults (*n* = 11) showed that upon introduction of BSMs, patients with bipolar disorder had an improvement of mood symptoms while simultaneously clinically managed on fewer psychotropic medications (more than a 50% reduction). This may indicate that when taken adjunctively, BSM may improve efficacy of psychiatric medication and lead to less polypharmacy and need for adjunctive medications in mood disorders (47). More studies are needed for this effect and to determine efficacy in cases of polypharmacy/treatment resistance.

### Psychosis

There is limited data on the use of BSM in psychosis. There is one case study of the use of BSM in the treatment of an 11 year old male diagnosed with psychosis NOS, OCD, GAD, SAD, and borderline intellectual functioning. He had multiple failed medication trials due to intolerable side effects and lack of efficacy. Beginning with a Children's Global Assessment Scale (CGAS) score of 35, by 14 months of BSM treatment his CGAS score was 75 with remittance of psychosis, OCD, GAD, SAD (48). In adults, an open label study followed 19 adults with psychotic disorders for two years. At the end of the two years, when compared to a group who chose treatment as usual, those in the BSM group reported fewer psychotic symptoms, required smaller doses of medication to be effective, and had fewer medication side effects (49).

### Substance Use

In a 12-week RCT, individuals with nicotine dependence (*n* = 107) were assigned to either a placebo group (*n* = 50) or a micronutrient group (*n* = 57); (50). The groups did not differ on the primary outcome of continuous abstinence at 12 weeks using intention-to-treat analysis (18% for placebo vs. 28% for micronutrient group, CI: 0.71–4.48). However, participants in the micronutrient group reported reduced cigarette consumption throughout the trial.

In a subset of participants who successfully completed the trial, the cessation rate was 42% in the micronutrient group compared to 23% in the placebo group (OR = 2.44). Notably, the effect of BSMs on cessation resembled the effectiveness of commonly prescribed medications for nicotine cessation, such as bupropion (odds ratio = 2.13) or varenicline (odds ratio = 2.88) (51). There were no notable differences in side effect occurrence between the micronutrient and placebo groups. The authors postulated that the underlying mechanism behind the positive impacts of BSMs on smoking cessation could potentially involve addressing neurobiological changes that stem from nicotine withdrawal and its associated neurochemical effects (e.g., nicotine's interaction with acetylcholine receptors and elevation of dopamine levels in the brain).

### Additional clinical considerations

Numerous investigations have been conducted on the efficacy of various micronutrient formulations. However, two in particular, namely Daily Essential Nutrients and EMPowerPlus, have garnered significant attention due to their recurrent examination across diverse medical contexts, availability for purchase, favorable safety profile (52), and research from independent scientists. Utilizing these interventions in a clinical setting necessitates adequate training and education, as the BSMs have contraindications and interact with psychotropic medications (22, 23). The most common barriers for patients include the requirement for multiple pills per day (4–12 depending on age and formulation) and cost ($50–150/month), as well as lack of insurance coverage.

## Discussion

Despite the availability of an expansive array of pharmacotherapeutic and psychotherapeutic treatment options, it is concerning that the prevalence of anxiety and depression is increasing.

This challenge offers the field of psychiatry a unique vantage point to investigate latent etiological factors that remain unaddressed. Within this context, the notion of nutritional insufficiencies as potential catalysts for the persistence or exacerbation of mental health disorders becomes a salient point of inquiry. Consequently, nutritional-based interventions are a pertinent avenue for exploration.

The phenomenon of irritability and emotional dysregulation is pervasive across various DSM-5 diagnostic categories and holds transdiagnostic relevance. Particularly within pediatric populations, antipsychotic medications represent the only treatment with strong support for the treatment of irritability, and this is specifically when irritability is presenting as a core feature of autism spectrum disorder (ASD) (53). However, antipsychotic medications have substantial endocrine, metabolic, and neurologic side effects. There is a large gap and need for further treatments to address pediatric irritability (54).

There is considerable evidence that BSMs can improve emotional regulation, irritability, and global functioning with ADHD being the psychiatric diagnosis most studied. There is some evidence that they can also improve measures of anxiety and stress, both as a function of a mental illness and as a reaction to a traumatic event.

In light of the pressing imperative for nonpharmacological alternatives in addressing pediatric mental health, there exists a compelling rationale for the further investigation and clinical attention for the use of BSMs.

## Data Availability

The original contributions presented in the study are included in the article/Supplementary Material, further inquiries can be directed to the corresponding author.
